# Supplementary material for: A descriptive analysis of the data availability statements accompanying medRxiv preprints and a comparison with their published counterparts
Source: PLoS One. 2021 May 13;16(5):e0250887. doi: 10.1371/journal.pone.0250887 (PMC8118451; doi:10.1371/journal.pone.0250887)
Supplement: S1 File — (DOCX) [file pone.0250887.s001.docx]

S1 File

Table of Contents

[1 Supplementary Tables 2](#_Toc70428358)

[1.1 Supplementary Table 1 2](#_Toc70428359)

[1.2 Supplementary Table 2 3](#_Toc70428360)

[1.3 Supplementary Table 3 9](#_Toc70428361)

[1.4 Supplementary Table 4 10](#_Toc70428362)

[2 Supplementary Materials 11](#_Toc70428363)

[2.1 Supplementary Material 1 11](#_Toc70428364)

# Supplementary Tables

## S1 Table

Table 1: Change in data availability openness, stratified by preprint decision category and subsequently by journal open data policy.

| **Preprint decision** | **Open data policy** | **Number of journals  (N)** | **Number of records  (N)** | **Open at preprint  % (N)** | **Open at publication  % (N)** | **Change from preprint to publication** | | |
| --- | --- | --- | --- | --- | --- | --- | --- | --- |
|  |  |  |  |  |  | **More open  (N)** | **More closed  (N)** | **No change  (N)** |
| **(1) Data not made available** | Does not mandate open data | 12 | 13 | 0% (0) | 23.1% (3) | 3 | 0 | 10 |
|  | Mandates open data | 3 | 7 | 0% (0) | 28.6% (2) | 2 | 0 | 5 |
| **(2) Data available on request to authors** | Does not mandate open data | 31 | 33 | 0% (0) | 9.1% (3) | 3 | 0 | 30 |
|  | Mandates open data | 5 | 11 | 0% (0) | 63.6% (7) | 7 | 0 | 4 |
| **(3) Data will be made available in the future (link provided)** | Does not mandate open data | 2 | 2 | 0% (0) | 50% (1) | 1 | 0 | 1 |
|  | Mandates open data | 1 | 1 | 0% (0) | 0% (0) | 0 | 0 | 1 |
| **(4) Data will be made available in the future (no link provided)** | Does not mandate open data | 4 | 4 | 0% (0) | 75% (3) | 3 | 0 | 1 |
|  | Mandates open data | 3 | 3 | 0% (0) | 66.7% (2) | 2 | 0 | 1 |
| **(5) Data available from central repository (access-controlled or open access), but insufficient detail available to find specific dataset** | Does not mandate open data | 17 | 21 | 0% (0) | 0% (0) | 0 | 0 | 21 |
|  | Mandates open data | 8 | 15 | 0% (0) | 33.3% (5) | 5 | 0 | 10 |
| **(6) Data available from central access-controlled repository, and sufficient details included to identify specific dataset e.g. via extract or accession ID or date stamp** | Does not mandate open data | 2 | 2 | 0% (0) | 0% (0) | 0 | 0 | 2 |
|  | Mandates open data | 1 | 1 | 0% (0) | 0% (0) | 0 | 0 | 1 |
| **(7) Data available in the manuscript/supplementary files** | Does not mandate open data | 9 | 9 | 100% (9) | 55.6% (5) | 0 | 4 | 5 |
|  | Mandates open data | 2 | 8 | 100% (8) | 100% (8) | 0 | 0 | 8 |
| **(8) Data available via a online repository that is not access-controlled e.g. Dryad, Zenodo** | Does not mandate open data | 10 | 10 | 100% (10) | 60% (6) | 0 | 4 | 6 |
|  | Mandates open data | 5 | 11 | 100% (11) | 100% (11) | 0 | 0 | 11 |

## S2 Table

Table 2: Change in data availability statement openness, stratified by journal.

| **Journal** | **Open data policy** | **Number of records  (N)** | **Open at preprint  % (N)** | **Open at publication  % (N)** | **Change from preprint to publication** | | |
| --- | --- | --- | --- | --- | --- | --- | --- |
|  |  |  |  |  | **More open  (N)** | **More closed  (N)** | **No change  (N)** |
| **Acta Neuropathologica Communications** | Does not mandate open data | 1 | 0% (0) | 0% (0) | 0 | 0 | 1 |
| **Alzheimer's Research & Therapy** | Mandates open data | 1 | 0% (0) | 0% (0) | 0 | 0 | 1 |
| **Annals of the Rheumatic Diseases** | Does not mandate open data | 2 | 0% (0) | 50% (1) | 1 | 0 | 1 |
| **Archives of Public Health** | Does not mandate open data | 1 | 0% (0) | 0% (0) | 0 | 0 | 1 |
| **Asia-Pacific Journal of Ophthalmology** | Does not mandate open data | 1 | 0% (0) | 0% (0) | 0 | 0 | 1 |
| **BMC Cancer** | Does not mandate open data | 2 | 50% (1) | 50% (1) | 1 | 1 | 0 |
| **BMC Endocrine Disorders** | Does not mandate open data | 1 | 0% (0) | 0% (0) | 0 | 0 | 1 |
| **BMC Medicine** | Does not mandate open data | 2 | 50% (1) | 50% (1) | 1 | 1 | 0 |
| **BMC Ophthalmology** | Does not mandate open data | 1 | 100% (1) | 0% (0) | 0 | 1 | 0 |
| **BMC Pediatrics** | Does not mandate open data | 1 | 0% (0) | 100% (1) | 1 | 0 | 0 |
| **BMC Public Health** | Does not mandate open data | 1 | 100% (1) | 100% (1) | 0 | 0 | 1 |
| **BMJ** | Does not mandate open data | 2 | 50% (1) | 0% (0) | 0 | 1 | 1 |
| **BMJ Global Health** | Does not mandate open data | 3 | 0% (0) | 33.3% (1) | 1 | 0 | 2 |
| **BMJ Open** | Does not mandate open data | 3 | 33.3% (1) | 33.3% (1) | 1 | 1 | 1 |
| **BMJ Open Quality** | Does not mandate open data | 1 | 0% (0) | 0% (0) | 0 | 0 | 1 |
| **Cell** | Mandates open data | 1 | 0% (0) | 0% (0) | 0 | 0 | 1 |
| **Cell Discovery** | Mandates open data | 1 | 0% (0) | 0% (0) | 0 | 0 | 1 |
| **Clinical Epigenetics** | Does not mandate open data | 2 | 0% (0) | 0% (0) | 0 | 0 | 2 |
| **Clinical Infectious Diseases** | Does not mandate open data | 1 | 0% (0) | 0% (0) | 0 | 0 | 1 |
| **EBioMedicine** | Mandates open data | 1 | 0% (0) | 100% (1) | 1 | 0 | 0 |
| **EClinicalMedicine** | Does not mandate open data | 1 | 0% (0) | 0% (0) | 0 | 0 | 1 |
| **Egyptian Journal of Medical Human Genetics** | Does not mandate open data | 1 | 100% (1) | 0% (0) | 0 | 1 | 0 |
| **Epidemics** | Does not mandate open data | 1 | 100% (1) | 100% (1) | 0 | 0 | 1 |
| **Epidemiology** | Does not mandate open data | 1 | 0% (0) | 0% (0) | 0 | 0 | 1 |
| **ESMO Open** | Does not mandate open data | 1 | 0% (0) | 0% (0) | 0 | 0 | 1 |
| **European Psychiatry** | Does not mandate open data | 2 | 0% (0) | 0% (0) | 0 | 0 | 2 |
| **European Respiratory Journal** | Does not mandate open data | 1 | 0% (0) | 0% (0) | 0 | 0 | 1 |
| **Eurosurveillance** | Does not mandate open data | 1 | 100% (1) | 100% (1) | 0 | 0 | 1 |
| **Experimental Eye Research** | Does not mandate open data | 1 | 0% (0) | 0% (0) | 0 | 0 | 1 |
| **F1000Research** | Mandates open data | 4 | 0% (0) | 75% (3) | 3 | 0 | 1 |
| **Family Practice** | Does not mandate open data | 1 | 100% (1) | 100% (1) | 0 | 0 | 1 |
| **Frontiers in Bioengineering and Biotechnology** | Does not mandate open data | 1 | 0% (0) | 0% (0) | 0 | 0 | 1 |
| **Frontiers in Immunology** | Does not mandate open data | 1 | 0% (0) | 0% (0) | 0 | 0 | 1 |
| **Frontiers in Integrative Neuroscience** | Does not mandate open data | 1 | 0% (0) | 0% (0) | 0 | 0 | 1 |
| **Frontiers in Medicine** | Does not mandate open data | 2 | 0% (0) | 0% (0) | 0 | 0 | 2 |
| **Frontiers in Neurology** | Does not mandate open data | 1 | 0% (0) | 0% (0) | 0 | 0 | 1 |
| **Frontiers in Nutrition** | Does not mandate open data | 1 | 100% (1) | 100% (1) | 0 | 0 | 1 |
| **Frontiers in Psychiatry** | Does not mandate open data | 1 | 0% (0) | 0% (0) | 0 | 0 | 1 |
| **Frontiers in Public Health** | Does not mandate open data | 2 | 50% (1) | 50% (1) | 0 | 0 | 2 |
| **Gates Open Research** | Mandates open data | 1 | 0% (0) | 0% (0) | 0 | 0 | 1 |
| **Genetics** | Does not mandate open data | 1 | 0% (0) | 0% (0) | 0 | 0 | 1 |
| **Genetics in Medicine** | Does not mandate open data | 1 | 0% (0) | 0% (0) | 0 | 0 | 1 |
| **Genome Medicine** | Does not mandate open data | 2 | 50% (1) | 50% (1) | 0 | 0 | 2 |
| **Human Brain Mapping** | Does not mandate open data | 1 | 0% (0) | 0% (0) | 0 | 0 | 1 |
| **Human Molecular Genetics** | Does not mandate open data | 1 | 100% (1) | 0% (0) | 0 | 1 | 0 |
| **Implementation Science Communications** | Does not mandate open data | 1 | 0% (0) | 0% (0) | 0 | 0 | 1 |
| **International Journal of Behavioral Nutrition and Physical Activity** | Does not mandate open data | 1 | 0% (0) | 0% (0) | 0 | 0 | 1 |
| **International Journal of Cancer** | Does not mandate open data | 1 | 0% (0) | 0% (0) | 0 | 0 | 1 |
| **International Journal of Health Geographics** | Does not mandate open data | 2 | 0% (0) | 0% (0) | 0 | 0 | 2 |
| **International Journal of Infectious Diseases** | Mandates open data | 2 | 0% (0) | 0% (0) | 0 | 0 | 2 |
| **JAMA Network Open** | Does not mandate open data | 1 | 0% (0) | 0% (0) | 0 | 0 | 1 |
| **Journal of Clinical Medicine** | Mandates open data | 1 | 0% (0) | 0% (0) | 0 | 0 | 1 |
| **Journal of Clinical Pathology** | Does not mandate open data | 1 | 0% (0) | 0% (0) | 0 | 0 | 1 |
| **Journal of Experimental Orthopaedics** | Does not mandate open data | 1 | 0% (0) | 100% (1) | 1 | 0 | 0 |
| **Journal of Infection** | Does not mandate open data | 1 | 0% (0) | 0% (0) | 0 | 0 | 1 |
| **Journal of Medical Internet Research** | Does not mandate open data | 1 | 100% (1) | 0% (0) | 0 | 1 | 0 |
| **Journal of Neurology, Neurosurgery & Psychiatry** | Does not mandate open data | 1 | 0% (0) | 0% (0) | 0 | 0 | 1 |
| **Journal of Oncology** | Does not mandate open data | 1 | 0% (0) | 0% (0) | 0 | 0 | 1 |
| **Journal of Pregnancy** | Does not mandate open data | 1 | 0% (0) | 100% (1) | 1 | 0 | 0 |
| **Journal of The Royal Society Interface** | Mandates open data | 1 | 0% (0) | 0% (0) | 0 | 0 | 1 |
| **Journal of Virology** | Mandates open data | 1 | 100% (1) | 100% (1) | 0 | 0 | 1 |
| **mBio** | Mandates open data | 1 | 0% (0) | 100% (1) | 1 | 0 | 0 |
| **Molecular & Cellular Proteomics** | Mandates open data | 1 | 0% (0) | 0% (0) | 0 | 0 | 1 |
| **Molecular Autism** | Does not mandate open data | 1 | 0% (0) | 0% (0) | 0 | 0 | 1 |
| **Multiple Sclerosis Journal** | Does not mandate open data | 1 | 0% (0) | 0% (0) | 0 | 0 | 1 |
| **Nature Machine Intelligence** | Does not mandate open data | 1 | 0% (0) | 0% (0) | 0 | 0 | 1 |
| **Nature Medicine** | Does not mandate open data | 4 | 0% (0) | 0% (0) | 0 | 0 | 4 |
| **NeuroImage: Clinical** | Does not mandate open data | 2 | 0% (0) | 0% (0) | 0 | 0 | 2 |
| **Neurology** | Does not mandate open data | 2 | 0% (0) | 50% (1) | 1 | 0 | 1 |
| **Neurology - Neuroimmunology Neuroinflammation** | Does not mandate open data | 1 | 100% (1) | 100% (1) | 0 | 0 | 1 |
| **Occupational and Environmental Medicine** | Does not mandate open data | 1 | 0% (0) | 100% (1) | 1 | 0 | 0 |
| **PeerJ** | Mandates open data | 1 | 0% (0) | 100% (1) | 1 | 0 | 0 |
| **PLOS Computational Biology** | Mandates open data | 2 | 50% (1) | 100% (2) | 1 | 0 | 1 |
| **PLOS Medicine** | Mandates open data | 1 | 0% (0) | 100% (1) | 1 | 0 | 0 |
| **PLOS Neglected Tropical Diseases** | Mandates open data | 2 | 50% (1) | 50% (1) | 0 | 0 | 2 |
| **PLOS ONE** | Mandates open data | 31 | 41.9% (13) | 67.7% (21) | 8 | 0 | 23 |
| **PLOS Pathogens** | Mandates open data | 1 | 100% (1) | 100% (1) | 0 | 0 | 1 |
| **Prostate Cancer** | Does not mandate open data | 1 | 0% (0) | 0% (0) | 0 | 0 | 1 |
| **Psychiatry Research** | Does not mandate open data | 1 | 0% (0) | 0% (0) | 0 | 0 | 1 |
| **Respiratory Research** | Does not mandate open data | 1 | 0% (0) | 0% (0) | 0 | 0 | 1 |
| **Science** | Does not mandate open data | 1 | 0% (0) | 0% (0) | 0 | 0 | 1 |
| **Scientific Data** | Mandates open data | 1 | 0% (0) | 0% (0) | 0 | 0 | 1 |
| **Scientific Reports** | Does not mandate open data | 6 | 16.7% (1) | 16.7% (1) | 0 | 0 | 6 |
| **Supportive Care in Cancer** | Does not mandate open data | 1 | 0% (0) | 0% (0) | 0 | 0 | 1 |
| **Swiss Medical Weekly** | Does not mandate open data | 1 | 100% (1) | 100% (1) | 0 | 0 | 1 |
| **The British Journal of Psychiatry** | Does not mandate open data | 1 | 0% (0) | 0% (0) | 0 | 0 | 1 |
| **The Lancet Infectious Diseases** | Does not mandate open data | 1 | 100% (1) | 100% (1) | 0 | 0 | 1 |
| **Translational Psychiatry** | Does not mandate open data | 1 | 0% (0) | 0% (0) | 0 | 0 | 1 |
| **Vaccine** | Does not mandate open data | 1 | 0% (0) | 0% (0) | 0 | 0 | 1 |
| **Wellcome Open Research** | Mandates open data | 2 | 100% (2) | 100% (2) | 0 | 0 | 2 |

## S3 Table

Table 3: Categories for the 22 records that were not open despite being published in a journal that requires open data.

| **Key** | **Sub-category** | **Number of records** |
| --- | --- | --- |
| **1** | Data not made available | 7 |
| **2** | Data available on request to authors | 3 |
| **5** | Data available from central repository (access-controlled or open access), but insufficient detail available to find specific dataset | 8 |
| **6** | Data available from central access-controlled repository, and sufficient details included to identify specific dataset e.g. via extract or accession ID or date stamp | 4 |

## S4 Table

Table 4: Comparison of code availability declarations between data availability statements and full text manuscripts.

|  | | **Full text** | |
| --- | --- | --- | --- |
|  | **_** | **Code availability described** | **Code availability not described** |
| **Data availability statement** | **Code availability described** | 53 | 16 |
|  | **Code availability not described** | 22 | 309 |

# Supplementary Materials

## Supplementary Material 1

For items that met the criteria for multiple categories (e.g. “Most of the data analyzed in this manuscript are provided either within the manuscript itself, or in the manuscript posted by Sasani et al. on bioRxiv at <https://www.biorxiv.org/content/10.1101/552117v2> and its accompanying links; additional data may be accessed by contacting the corresponding author (Dr. Cawthon).”), we took a conservative approach, and categorized them based on the least permissive aspect of the statement. This approach was taken on the basis that ***all*** data must be available - for example, in the above statement, the additional information that is available on request may be required in order to reproduce the analysis.
