# Supplementary material for: A descriptive analysis of the data availability statements accompanying medRxiv preprints and a comparison with their published counterparts
Source: PLoS One. 2021 May 13;16(5):e0250887. doi: 10.1371/journal.pone.0250887 (PMC8118451; doi:10.1371/journal.pone.0250887)
Supplement: Attachment — Submitted filename: Response to Reviewers.docx [file pone.0250887.s002.docx]

| Reviewer: 1 | | |
| --- | --- | --- |
| 1.1 | The methodological part description should be improved to facilitate the understanding. | Following the reviewer’s comments, we have extensively restructured the methods section of the manuscript to better group related methodological aspects and provide an improved flow for the reader.  We have also attempted to better standardize our language across the manuscript, for example using “mandated” consistently (rather than a mix of “mandated” and “required”) when describing journal’s data sharing policies. |
| 1.2 | The presentation and description of the results could be improved for a better understanding, including the tables. | In order to highlight the main findings of our study, we have sought to improve Table 1 by reducing the amount of information presented, improving the column labels, and adding additional formatting to make it easier to interpret. In addition, we have re-written the paragraph describing our main findings in what we hope is clearer language.  We have additionally moved the table presenting the results of the code availability section to the Supplementary Material (now exists as Supp Table 4), as while this is useful to have, this table does not add much to the main manuscript beyond that presented in the text.  Finally, we have reordered the legend in the main figure to make it easier for readers to interpret. |
| 1.3 | I would like to suggest the replacement of the terms “more open” and “more closed” for more suitable terms. | We have chosen to retain these terms as, despite conversation with our colleagues and others working in the field, we have not been able to identify alternative phrasing that captures our precise meaning as well as these phrases. However, we acknowledge that the terms are not intuitive and have added detailed explanation of these terms to the “Changes between preprinted and published statements” section in the Methods. |
| 1.4 | The results on the abstract must be revised. Some values are different from those showed on the results section. Also, there is a sentence that is not correct. Please, revise. | We thank the reviewer for highlighting this discrepancy between the abstract and the results, which was intentional but confusing. We have now reworded the Abstract to ensure consistency between the two sections. |
| Reviewer: 2 | | |
| 2.1 | My only suggestion would be that it might be interesting to discuss the number of papers that are NOT open despite being published in a journal that requires open data (22 out of 55 according to Table 2). Were these journals that just encouraged rather than required open data, or were papers published despite not following the policy? I would be very interested to know how almost half the papers in this category ended up not having open data. | We thank the reviewer for their useful feedback and kind comments on our manuscript.  We have now included an additional supplementary table (Supp Table 3) showing the breakdown of these 22 records across the categories defined in Table 1. In addition, we have included a new paragraph in the Results summarizing the reasons for these records being labelled as “closed” despite being published in a journal that required data sharing. |
