# Supplementary material for: A descriptive analysis of the data availability statements accompanying medRxiv preprints and a comparison with their published counterparts
Source: PLoS One. 2021 May 13;16(5):e0250887. doi: 10.1371/journal.pone.0250887 (PMC8118451; doi:10.1371/journal.pone.0250887)
Supplement: Attachment — Submitted filename: Response_Round2.docx [file pone.0250887.s003.docx]

| Editorial comments | | |
| --- | --- | --- |
| 1.1 | It is not clear how the data extraction related to the journals' data-sharing policies was performed. Please, clarify that. | We have added additional text to the Methods section (notably at the end of Section 4.2 and 4.3) to describe how journals’ data-sharing policies were extracted and assessed. We have also added text informing readers that a file containing the extracted polices are available for inspection in the repository associated with this manuscript. |
| 1.2 | The conclusion should be aligned with the objectives and results. Please, revise that. | We have reworked the Conclusion, moving some discursive material into the “Implications for policy” section, and adding new text that more accurately aligns with the objectives and results of our study. |
|  | | |
| 2.1 | Other changes | In the course of revising this manuscript to respond to the editorial comments above, we corrected some other small errors (for example, inconsistent use of “data-sharing policy” vs “data sharing policy”). These are all captured as tracked changes in the “plos-one-marked-up.docx” document. |
